# Supplementary material for: In vivo base editing rescues cone photoreceptors in a mouse model of early-onset inherited retinal degeneration
Source: Nat Commun. 2022 Apr 5;13:1830. doi: 10.1038/s41467-022-29490-3 (PMC8983734; doi:10.1038/s41467-022-29490-3)

SUPPLEMENTARY INFORMATION

In vivo base editing rescues cone photoreceptors in a mouse model of early-onset inherited retinal degeneration

Elliot H. Choi[1,2]†, Susie Suh[1,2]†*, Andrzej T. Foik[3], Henri Leinonen[1], Gregory A. Newby[4,5,6], Xin D. Gao[4,5,6], Samagya Banskota[4,5,6], Thanh Hoang[7], Samuel Du[1,8], Zhiqian Dong[1], Aditya Raguram[4,5,6], Sajeev Kohli[4,5,6], Seth Blackshaw[7], David C. Lyon[9], David R Liu[4,5,6], Krzysztof Palczewski[1,8,10,11]*

[1]Gavin Herbert Eye Institute, Department of Ophthalmology, University of California, Irvine, CA, USA

[2]Department of Pharmacology, Case Western Reserve University, Cleveland, OH, USA

[3]International Centre for Translational Eye Research, Polish Academy of Sciences, Warsaw, PL

[4]Merkin Institute of Transformative Technologies in Healthcare at Broad Institute of Harvard and MIT, Cambridge, MA, USA

[5]Department of Chemistry and Chemical Biology, Harvard University, Cambridge, MA, USA

[6]Howard Hughes Medical Institute, Harvard University, Cambridge, MA, USA

[7]Department of Neuroscience, Johns Hopkins University School of Medicine, Baltimore, MD, USA.

[8]Department of Physiology and Biophysics, University of California, Irvine, CA, USA

[9]Department of Anatomy and Neurobiology, School of Medicine, University of California, Irvine, CA, USA

[10]Department of Chemistry, University of California, Irvine, CA, USA

[11]Department of Molecular Biology and Biochemistry, University of California, Irvine, CA, USA

†These authors contributed equally, Elliot H. Choi, Susie Suh

*Corresponding authors, susie.suh@case.edu; kpalczew@uci.edu

Table of Contents

Supplementary Fig. 1. Base editing analysis of *Rpe65* in mouse RPE genomic DNA following *in vivo* lentiviral treatment. 3

Supplementary Fig. 2. Potential off-target sites of NG-ABE and sgRNA A6 identified by CIRCLE-seq 4

Supplementary Fig. 3. RPE65 rescue in *rd12* mice treated with LV-NG-ABE-A6 5

Supplementary Fig. 4. Use of dual-AAV vectors for split base editor delivery 6

Supplementary Fig. 5. Representative retinal flatmount of 2-month-old *Gnat1*$^{-/-}$ mouse 7

Supplementary Fig. 6. Comparison of photopic ERG and cone count to control *Gnat1*$^{-/-}$ mice 8

Supplementary Fig. 7. Long-term protection of cone function and structure by ABE treatment in 6-month-old *rd12Gnat1*$^{-/-}$ mice 10

Supplementary Fig. 8. Heatmap of scRNA-seq correlation between each sample by Pearson's correlation coefficient analysis 11

Supplementary Fig. 9. Dot plot of transcript expression for genes associated with cell survival, death or stress response in cone cells across three groups. 12

Supplementary Table 1. Average gene expression level in cone cells of treated, untreated, and WT retinas. 13

Supplementary Table 2. List of top differentially expressed genes (FDR <0.05) between treated, untreated and wildtype cone cells. 14

Supplementary Table 3. Primers used for off-target analysis. 16

Original uncropped blots 17

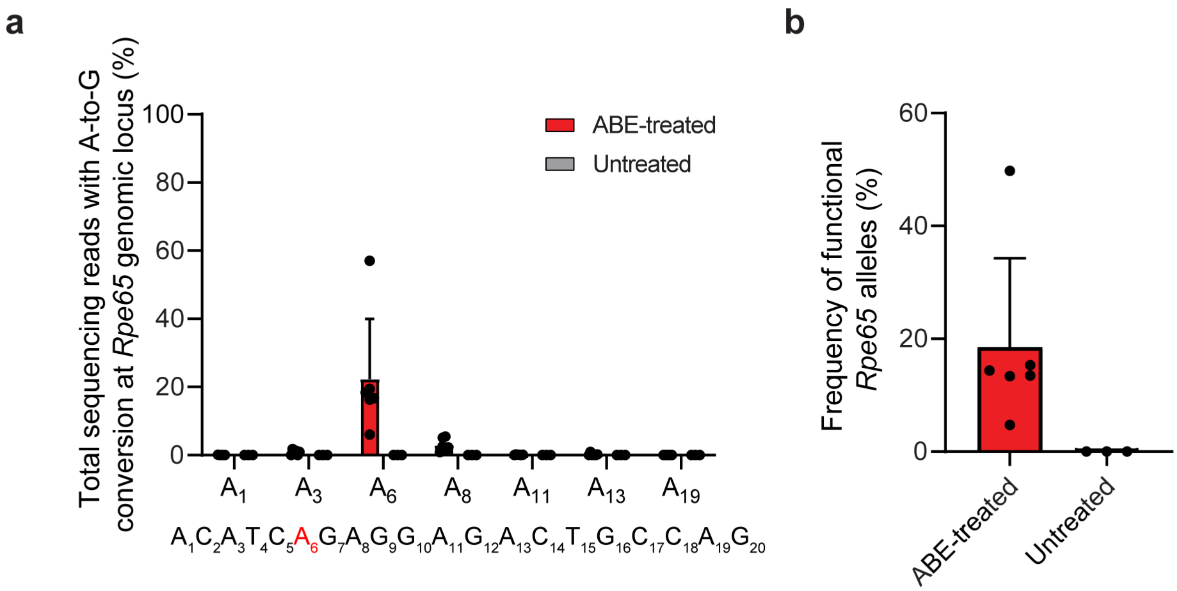

Supplementary Fig. 1. Base editing analysis of *Rpe65* in mouse RPE genomic DNA following *in vivo* lentiviral treatment. a, Frequency of A-to-G conversion in the *Rpe65* gDNA isolated from lentivirus-injected (ABE-treated) and PBS-injected (untreated) *rd12* mouse eyes. Bottom sequence represents 20-nucleotide sgRNA-A6 with the targeted mutation highlighted in red. ABE-treated, n = 6; Untreated, n = 3. Mean ± SD. **b**, Frequency of precisely corrected, functional *Rpe65* alleles from the same eyes in (A)***.***

a

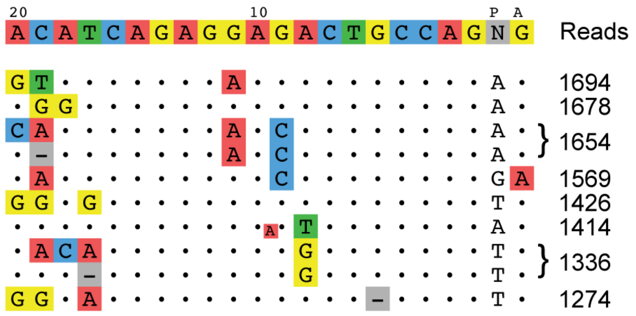

b

Name	Chromosome	Start	End	Closest gene	Distance
OT1	chr4	63653116	63653138	Gm11214 (pseudogene)	9 kb downstream
OT2	chr5	61764466	61764488	Gm4038 (lncRNA)	on lncRNA
OT3	chr2	67928115	67928137	B3galt1	intronic
OT4	chr5	136542721	136542743	Cux1	intronic
OT5	chr10	64026020	64026042	Ctnna3	intronic
OT6	chr1	96841942	96841965	Slco4c1	intronic
OT7	chr1	181236170	181236192	Gm38664 (mRNA predicted pseudogene)	on pseudogene exon
OT8	chr9	112436846	112436867	Gm39431	22 kb upstream
OT9	chr13	72859056	72859077	D730050B12Rik (lncRNA)	on lncRNA
OT10	chr2	5584558	5584579	Camk1d	intronic

Supplementary Fig. 2. Potential off-target sites of NG-ABE and sgRNA A6 identified by CIRCLE-seq. a, Alignments of the top 10 off-target sequences identified by CIRCLE-seq using NG-Cas9 and sgRNA-A6. The read count of the observed sequence is written at the right. Reference protospacer sequence is shown on top. Nucleotides that match the protospacer are indicated with a middle dot. Nucleotides that differ are shown for each site. Horizontal dashes indicate a skipped nucleotide in the alignment, and small nucleotide letters indicate an insertion relative to the protospacer. **b**, Location and identity of the 10 off-target sites shown in (**a**).

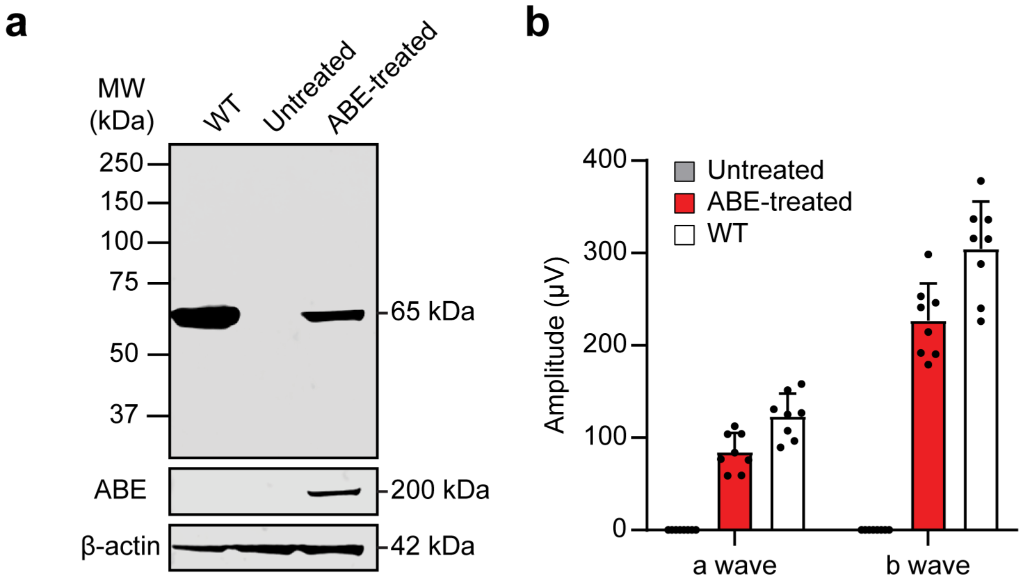

Supplementary Fig. 3. RPE65 rescue in *rd12* mice treated with LV-NG-ABE-A6. a, Immunoblot showing restoration of a full-length RPE65 (65 kDa) protein in the RPE tissue lysate from ABE-treated *rd12* mice. ABE (200 kDa), base editor; β-actin (42 kDa), loading control. **b,** Scotopic a-wave and b-wave amplitudes evoked with light stimulus of -0.3 log (cd•s/m^2) from age-matched WT, untreated and treated *rd12* mice. n = 8 eyes each group. Mean ± SD.

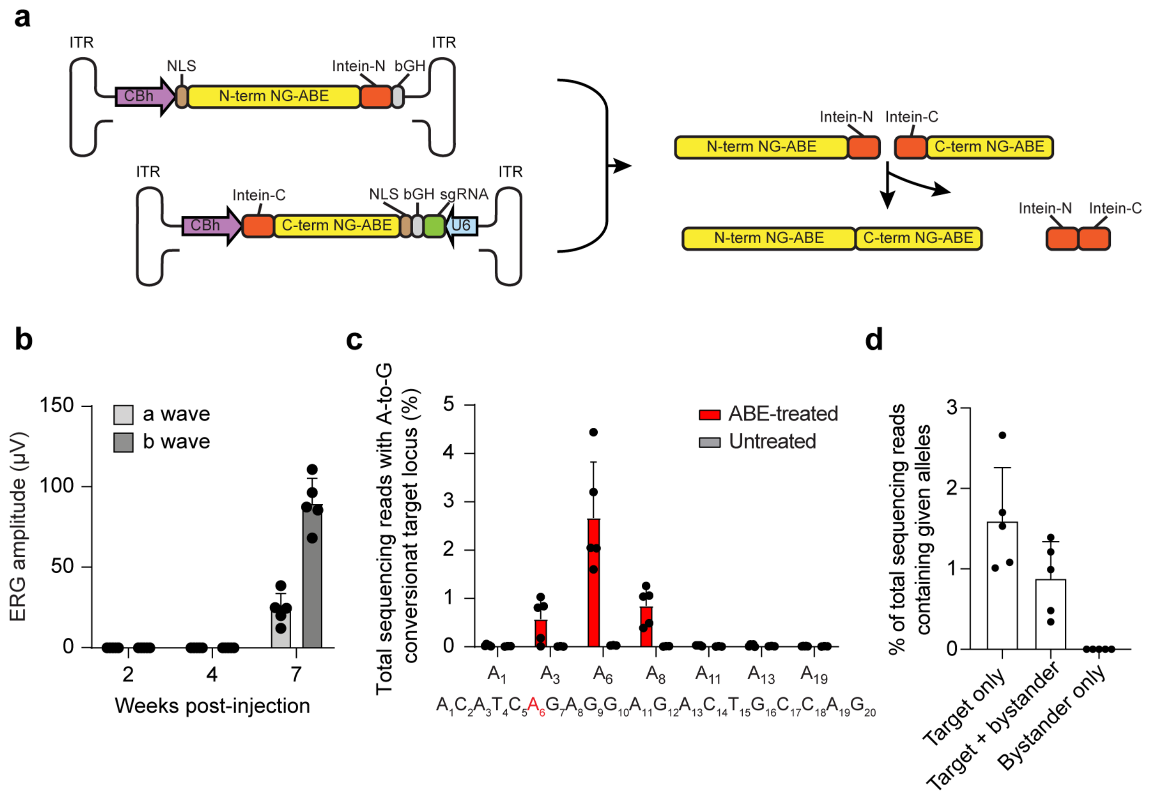

Supplementary Fig. 4. Use of dual-AAV vectors for split base editor delivery. a, Schematic diagram of split intein-AAV vectors. **b**, Scotopic ERG a-wave and b-wave amplitudes recorded at three timepoints in AAV-injected *rd12* mice. n = 5 eyes. Mean ± SD. **c**, Frequency of A-to-G conversion in the *Rpe65* genomic DNA isolated from AAV-injected and untreated *rd12* mouse eyes. Bottom sequence represents the 20-nucleotide sgRNA-A6 with the targeted mutation highlighted in red. ABE-treated, n = 5; Untreated, n = 3. Mean ± SD. **d**, Percentage of modified alleles in each sample used in (**c**).

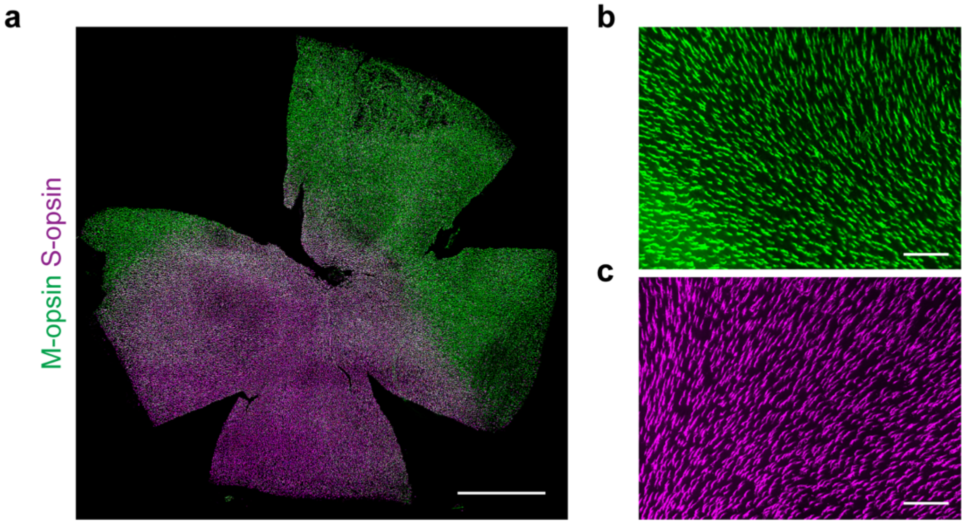

Supplementary Fig. 5. Representative retinal flatmount of 2-month-old *Gnat1*$^{-/-}$ mouse. a, Overall view of the retinal flatmount from a 2-month-old *Gnat1*$^{-/-}$ mouse, labelled with M-opsin (green) and S-opsin (purple) antibodies. Scale bar, 1 mm. **b**, Magnified view of M-cones labeled with M-opsin antibody (green) at the dorsal retina. Scale bar, 50 μm. **c**, Magnified view of S-cones labeled with S-opsin antibody (purple) at the ventral retina. Scale bar, 50 μm.

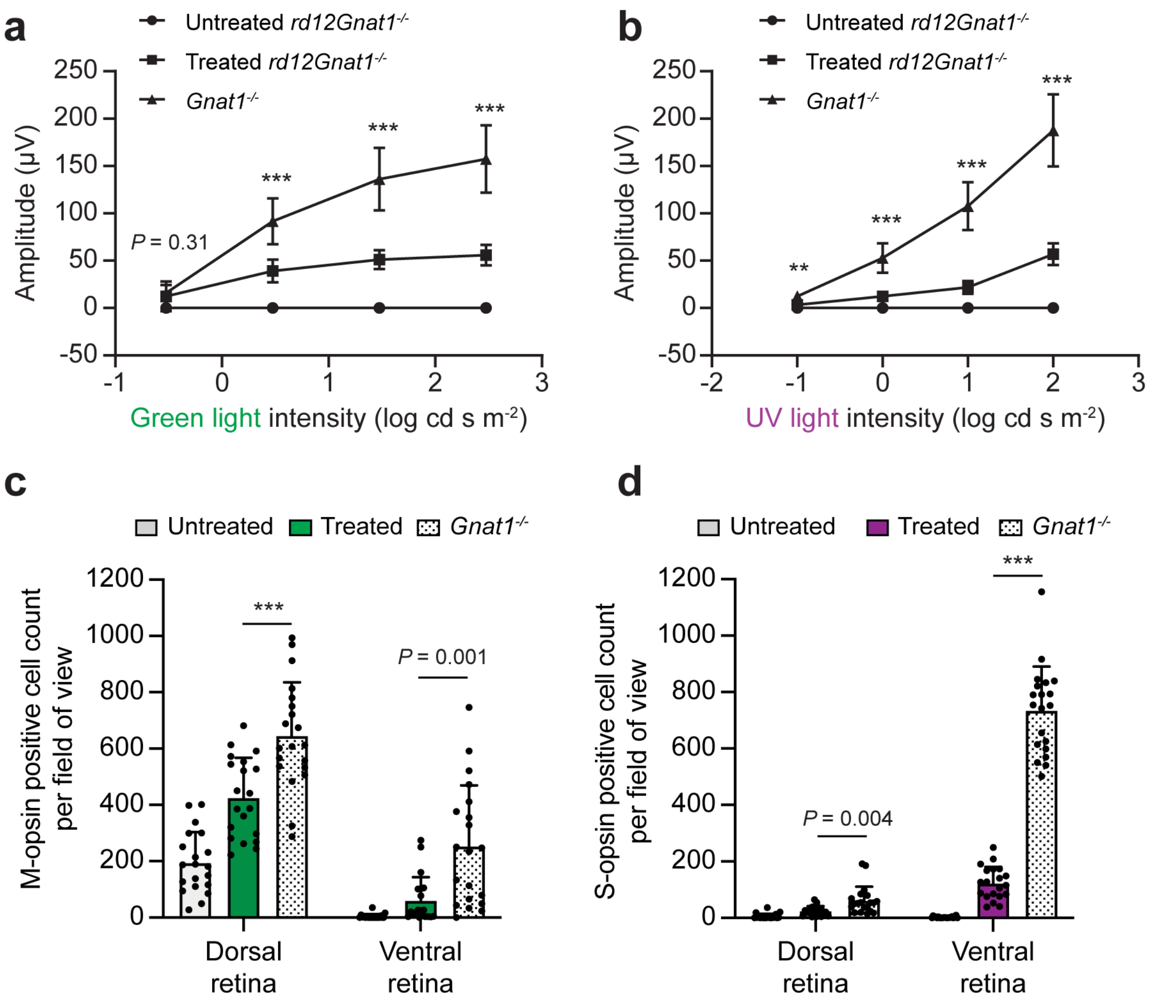

Supplementary Fig. 6. Comparison of photopic ERG and cone count to control *Gnat1*$^{-/-}$ mice. a, Photopic b-wave amplitudes evoked with green light flashes in untreated and treated *rd12Gnat1*$^{-/-}$ mice; and in *Gnat1*$^{-/-}$ mice. n = 8 mouse eyes, each group. Mean ± SD. ***, $P < 0.001$; two-tailed Mann-Whitney U-test for treated *rd12Gnat1*$^{-/-}$ vs. *Gnat1*$^{-/-}$. P values for 0.5, 1.5, and 2.5 light intensities are same at 0.0002. **b,** Photopic b-wave amplitudes evoked with UV light flashes in untreated and treated *rd12Gnat1*$^{-/-}$; and in *Gnat1*$^{-/-}$ mice. n = 8 mouse eyes, each group. Mean ± SD. **, $P < 0.01$; ***, $P < 0.001$; two-tailed Mann-Whitney U-test for treated *rd12Gnat1*$^{-/-}$ vs. *Gnat1*$^{-/-}$. P values are 0.002 at -1 and 0.0002 at 0, 1 and 2 UV light intensity. **c**, Quantification of M-cones in each quadrant at dorsal and ventral regions of the retina, 1 mm away from the optic nerve. Five quadrants across the dorsal or ventral retina were analyzed from each eye, with a total of 20 quadrants from 4 eyes each group. Mean ± SD. ***, $P < 0.001$; unpaired t-test, two-tailed. P value for dorsal retina is 0.00019. **d**, Quantification of S-cones in each quadrant at dorsal and ventral regions of the retina, 1 mm away from the optic nerve. Five quadrants across the dorsal or ventral retina were analyzed from each eye, with a total of 20 quadrants from 4 eyes each group. Mean ± SD. ***, $P < 0.001$; unpaired t-test, two-tailed. P value for ventral retina is < 0.000001.

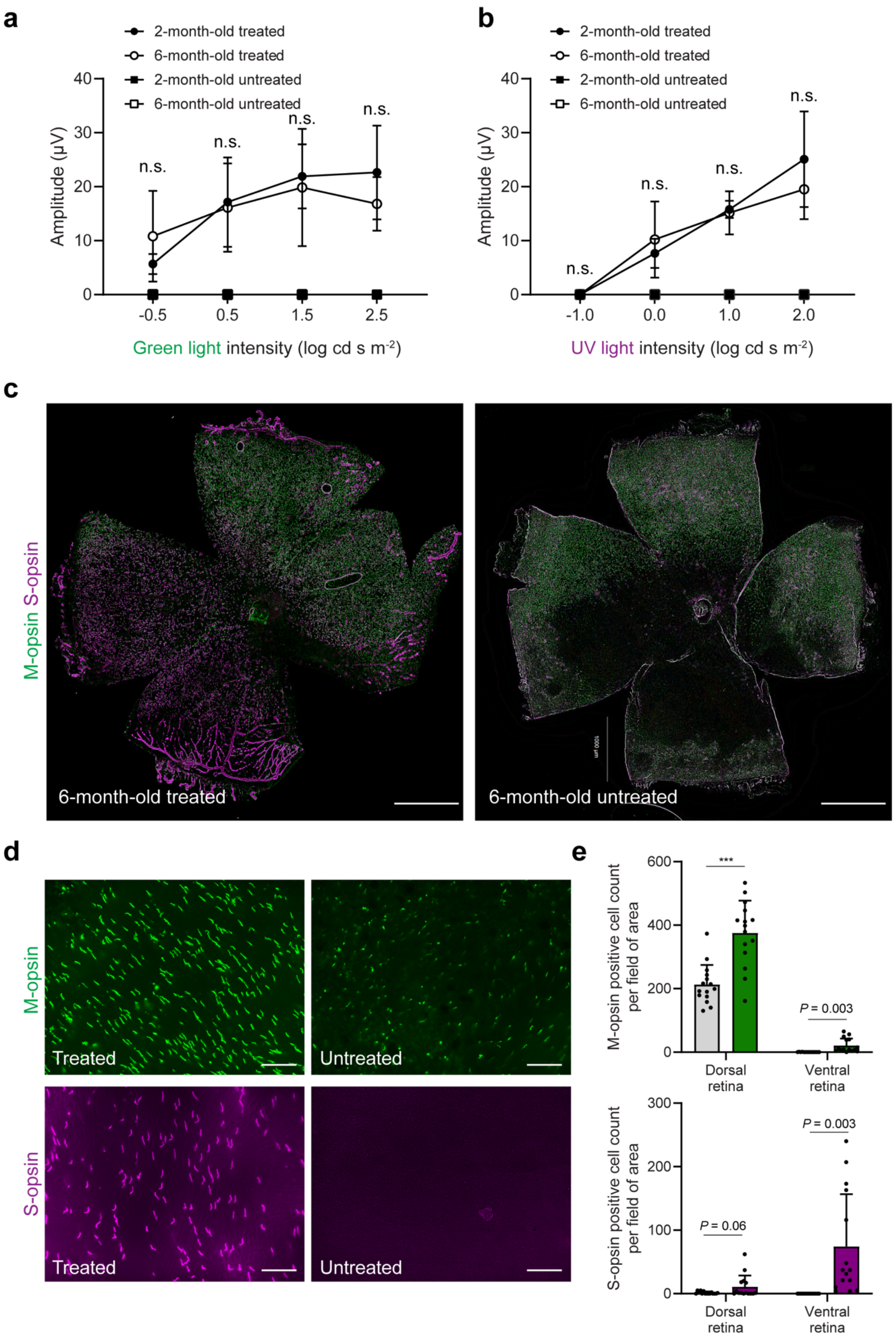
a
2-month-old treated
6-month-old treated
2-month-old untreated
6-month-old untreated
n.s.
Amplitude (µV)
0
10
20
30
40
-0.5
0.5
1.5
2.5
Green light intensity (log cd s m-2)
b
2-month-old treated
6-month-old treated
2-month-old untreated
6-month-old untreated
n.s.
Amplitude (µV)
-1.0
0.0
1.0
2.0
UV light intensity (log cd s m-2)
c
M-opsin S-opsin
6-month-old treated
6-month-old untreated
d
M-opsin
S-opsin
Treated
Untreated
e
M-opsin positive cell count per field of area
0
200
400
600
***
P = 0.003
Dorsal retina
Ventral retina
S-opsin positive cell count per field of area
0
100
200
300
P = 0.06
P = 0.003

Supplementary Fig. 7. Long-term protection of cone function and structure by ABE treatment in 6-month-old *rd12Gnat1$^{-/-}$* mice. a, Amplitudes of photopic ERG b-waves of M-cones recorded from the same eyes at 2 months and 6 months of age (n = 4 eyes). Mean ± SD. **b,** Amplitudes of photopic ERG b-waves of S-cones recorded from the same eyes at 2 months and 6 months of age (n = 4 eyes). Mean ± SD. **c,** Representative retinal flatmounts from 6-month-old treated (left) and untreated (right) *rd12Gnat1$^{-/-}$* mice, labeled with M-opsin (green) and S-opsin (purple) antibodies. Scale bar, 1 mm. **d**, Magnified view of M-cones (green) in dorsal, and S-cones (purple) in the ventral retina from treated and untreated *rd12Gnat1$^{-/-}$* mice. Scale bar, 50 μm. **e**, Quantification of M-cones (upper) and S-cones (lower) in each quadrant, as shown in (D), at the dorsal and ventral retina 1 mm away from the optic nerve. Five quadrants across the dorsal or ventral retina were analyzed from one eye, with a total of 15 quadrants from 3 eyes per group. Mean ± SD. ***, $P < 0.001$; unpaired t-test, two-tailed. P value for dorsal M-opsin is < 0.000001.

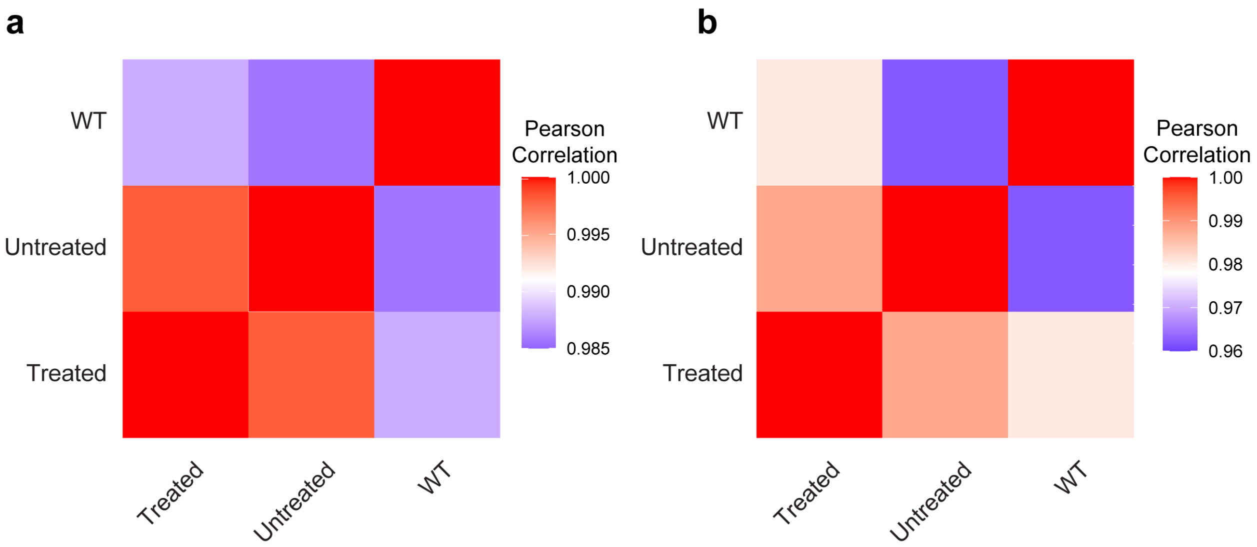

Supplementary Fig. 8. Heatmap of scRNA-seq correlation between each sample by Pearson's correlation coefficient analysis. a, cones. **b,** rods.

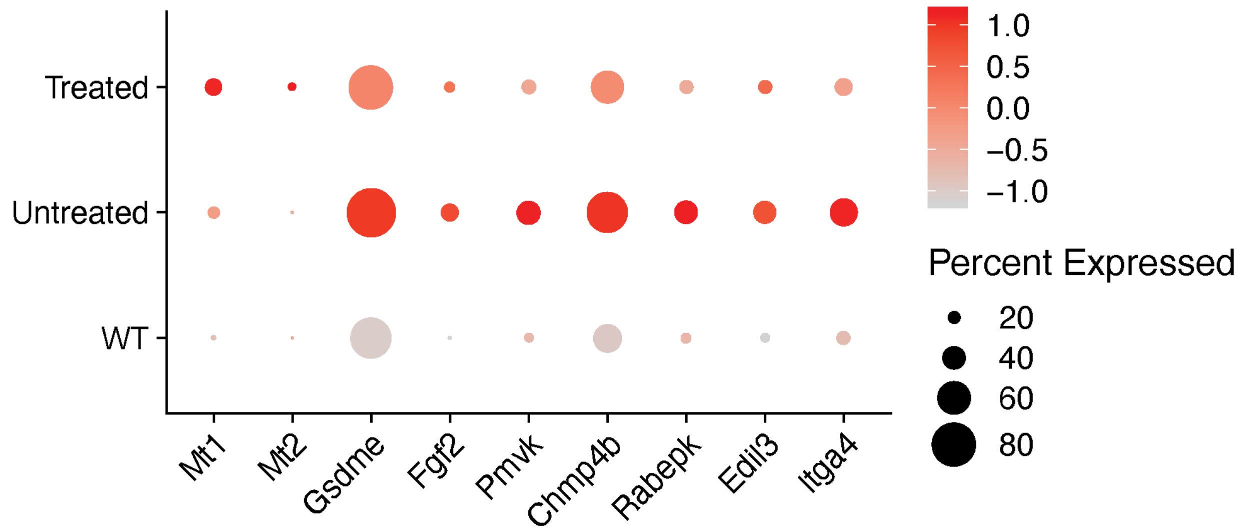

Supplementary Fig. 9. Dot plot of transcript expression for genes associated with cell survival, death or stress response in cone cells across three groups. *Mt1* and *Mt2* are candidate genes implicated in promoting photoreceptor survival. *Gsdme, Fgf2, Pmvk, Chmp4b, Rabepk, Edil3* and *Itga4* are candidate genes implicated in promoting photoreceptor cell death and stress response. The shades of red indicate average expression of each gene. The sizes of circles indicate percentage of the cells expressing each gene. All genes except *Mt2* are among top 48 differentially expressed genes identified at FDR < 0.05.

Supplementary Table 1. Average gene expression level in cone cells of treated, untreated, and WT retinas. Wilcoxon rank sum test was used for statistical analysis.

Gene	Protein	Treated	Untreated	WT	*P*-value (treated vs. untreated)
Opn1sw	S-opsin	27.4	2.4	61.3	1.18E-04
Opn1mw	M-opsin	61.2	55.6	43.8	n.s.
Arr3	Cone arrestin	58.4	34.6	60.4	3.16E-06
Rbp3	IRBP	28.2	20.0	28.1	6.43E-05
Gnat2	Gnat G(t) subunit alpha-2 (cone specific)	21.5	16.2	24.4	0.00199
Kcne2	Potassium voltage-gated channel subfamily E member 2	2.4	1.0	5.8	0.00172
Grk1	Rhodopsin kinase	4.9	3.6	6.2	0.02935

Supplementary Table 2. List of top differentially expressed genes (FDR <0.05) between treated, untreated and wildtype cone cells. Wilcoxon rank sum test was used for statistical analysis.

	p_val	avg_logFC	pct.1	pct.2	p_val_adj	cluster
**Fbxw13**	6.6E-10	0.259511058	0.23	0.042	2.13E-05	Treated
**Opn1sw**	1.7E-20	1.410933657	0.671	0.315	5.49E-16	WT
**Fbxw211**	4.28E-28	0.955148577	0.66	0.152	1.38E-23	Untreated
**Kcne2**	2.76E-21	0.932118588	0.738	0.391	8.9E-17	WT
**Gm17167**	7.5E-16	0.547956875	0.309	0.011	2.42E-11	WT
**Car8**	4.59E-15	0.551496592	0.639	0.242	1.48E-10	Untreated
**Ccdc136**	7.69E-15	0.921457332	0.682	0.44	2.48E-10	WT
**Mpp6**	4.86E-14	0.542563326	0.928	0.774	1.57E-09	Untreated
**Fgf2**	9.23E-13	0.267557435	0.299	0.056	2.98E-08	Untreated
**Junb1**	1.23E-12	0.68712041	0.753	0.395	3.97E-08	Untreated
**Vegfa**	1.03E-10	0.594456615	0.499	0.239	3.32E-06	WT
**F630040K05Rik**	1.31E-10	0.618330991	0.387	0.136	4.21E-06	WT
**Gnat2**	1.01E-09	0.252432225	0.972	0.962	3.27E-05	WT
**Pmvk**	2.29E-09	0.455065012	0.412	0.157	7.4E-05	Untreated
**Lars2**	4.2E-09	0.353012644	0.936	0.88	0.000135631	WT
**Gsdme**	4.75E-09	0.412614311	0.897	0.758	0.000153241	Untreated
**Fbxw21**	7.42E-09	0.504126641	0.494	0.195	0.000239707	Treated
**Syndig1l**	7.54E-09	0.463613387	0.306	0.092	0.000243537	WT
**Ppia1**	1.01E-08	0.436544254	0.845	0.738	0.000325111	Untreated
**Mt1**	2.65E-08	0.537405204	0.287	0.081	0.000856609	Treated
**Rpsa**	3.11E-08	0.365810776	0.825	0.742	0.001004874	Untreated
**mt-Nd1**	3.46E-08	0.293292028	0.959	0.942	0.001115718	Untreated
**Nap1l1**	1.04E-07	0.3595312	0.876	0.778	0.0033622	Untreated
**Rab6b**	1.2E-07	0.343048921	0.577	0.289	0.003864882	Untreated
**Fbxw131**	1.5E-07	0.25642428	0.196	0.045	0.004848502	Untreated
**Plk2**	1.7E-07	0.288493995	0.165	0.031	0.005492278	Untreated
**Itga4**	2.1E-07	0.331385053	0.495	0.233	0.006791926	Untreated
**Arr3**	2.32E-07	0.270589109	0.992	0.995	0.007500591	WT
**Tpt1**	2.62E-07	0.343074192	0.876	0.787	0.00844467	Untreated
**Cerkl**	2.96E-07	0.40040898	0.608	0.339	0.009559277	Untreated
**Rpl41**	3.47E-07	0.337053794	0.907	0.807	0.011198569	Untreated
**Atp2b1**	4.06E-07	0.294902969	0.948	0.845	0.013096801	Untreated
**Hmgb1**	4.37E-07	0.367012642	0.866	0.735	0.014122613	Untreated
**Edil3**	4.78E-07	0.263457967	0.392	0.157	0.015428256	Untreated
**Klhl33**	5.04E-07	0.487786714	0.448	0.288	0.016271048	WT
**Pdc**	5.67E-07	0.260429925	0.911	0.918	0.018319303	WT

**Rps16**	6.33E-07	0.368018491	0.866	0.7	0.020423119	Untreated
**Sgip1**	6.53E-07	0.263578401	0.911	0.853	0.02107044	WT
**Mfsd4a**	6.57E-07	0.330367651	0.443	0.202	0.021207391	Untreated
**Rgs9bp**	7.16E-07	0.399121346	0.741	0.674	0.02311086	WT
**mt-Co1**	7.24E-07	0.282103247	0.942	0.957	0.023375713	WT
**Rplp1**	7.57E-07	0.343610266	0.866	0.794	0.024452998	Untreated
**Chgb**	7.86E-07	0.352505064	0.856	0.796	0.025368842	Untreated
**Rabepk**	9.58E-07	0.326719312	0.402	0.17	0.030935991	Untreated
**Amer2**	1.1E-06	0.413519175	0.845	0.711	0.035534571	Untreated
**Ftl1**	1.22E-06	0.410101903	0.763	0.601	0.039508596	Untreated
**Chmp4b**	1.31E-06	0.353417033	0.742	0.52	0.042262555	Untreated
**Rabgef1**	1.32E-06	0.385182019	0.705	0.56	0.042629917	WT

Supplementary Table 3. Primers used for off-target analysis.

Name	Forward primer	Reverse primer
OT1	ACACTCTTTCCCTACACGACGCTCTTCCGATCTNNNNTTGCCAACAGGTTCTCCTATC	TGGAGTTCAGACGTGTGCTCTTCCGATCTCTGGCAGAACTGAACGAGG
OT2	ACACTCTTTCCCTACACGACGCTCTTCCGATCTNNNNGTGATCACAGAGGTGGGC	TGGAGTTCAGACGTGTGCTCTTCCGATCCATCAGTGACTTGGGATGATAGA
OT3	ACACTCTTTCCCTACACGACGCTCTTCCGATCTNNNNCCATCTGCCACCACTCAC	TGGAGTTCAGACGTGTGCTCTTCCGATCTAGTAGGCCTTTCAGGCATGA
OT4	ACACTCTTTCCCTACACGACGCTCTTCCGATCTNNNNGGAATGAGTCTGTGAGATCGC	TGGAGTTCAGACGTGTGCTCTTCCGATCCTCATTCTGTAGATCAGGCTGG
OT5	ACACTCTTTCCCTACACGACGCTCTTCCGATCTNNNNGCTAAGTCAGCCCTTCGTTG	TGGAGTTCAGACGTGTGCTCTTCCGATCTACCTCCCAGTGATGGGACT
OT6	ACACTCTTTCCCTACACGACGCTCTTCCGATCTNNNNTCTCACAGCAAATCACTGATCTA	TGGAGTTCAGACGTGTGCTCTTCCGATCTATCCTACTCAGTTCTGCAGATA
OT7	ACACTCTTTCCCTACACGACGCTCTTCCGATCTNNNNAAGTGGCCAACCGTGCGA	TGGAGTTCAGACGTGTGCTCTTCCGATCTTCTAACACTTCAATTCAGGTGG
OT8	ACACTCTTTCCCTACACGACGCTCTTCCGATCTNNNNCAGGACAAGTTGTCCCTGGC	TGGAGTTCAGACGTGTGCTCTTCCGATCGTGGGAGTGTTAAAGACAGCG
OT9	ACACTCTTTCCCTACACGACGCTCTTCCGATCTNNNNTGGCAGATAGGAGACACTTCT	TGGAGTTCAGACGTGTGCTCTTCCGATCCCTGATAAACCCTTCCTTGAC
OT10	ACACTCTTTCCCTACACGACGCTCTTCCGATCTNNNNGAATGCGGAAGACATGTTCTATTC	TGGAGTTCAGACGTGTGCTCTTCCGATCCATCAGAGGATTCCCAACGC

Original uncropped blots

Supplementary Fig. 3A

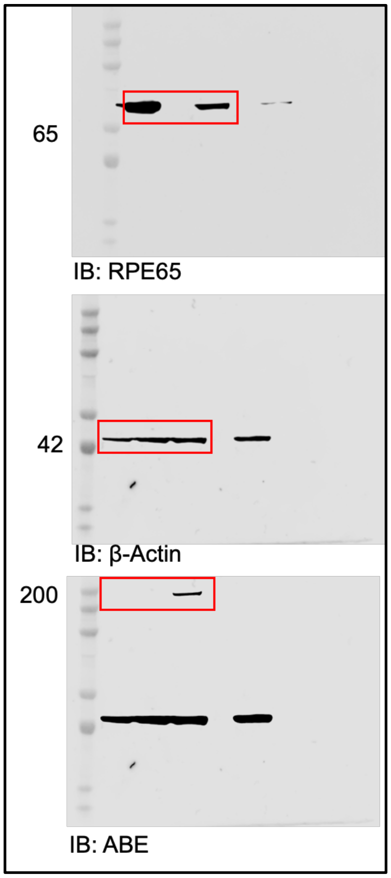

Supplement: Supplementary file 1 — Supplementary Information [file 41467_2022_29490_MOESM1_ESM.pdf]
