# Supplementary material for: In vivo base editing rescues cone photoreceptors in a mouse model of early-onset inherited retinal degeneration
Source: Nat Commun. 2022 Apr 5;13:1830. doi: 10.1038/s41467-022-29490-3 (PMC8983734; doi:10.1038/s41467-022-29490-3)
Supplement: Supplementary file 3 — Reporting Summary [file 41467_2022_29490_MOESM3_ESM.pdf]

Corresponding author(s): Susie Suh, Krzysztof Palczewski

Last updated by author(s): Feb 19, 2022

Reporting Summary

Nature Portfolio wishes to improve the reproducibility of the work that we publish. This form provides structure for consistency and transparency in reporting. For further information on Nature Portfolio policies, see our Editorial Policies and the Editorial Policy Checklist.

Statistics

For all statistical analyses, confirm that the following items are present in the figure legend, table legend, main text, or Methods section.

n/a	Confirmed	
☐	☒	The exact sample size (*n*) for each experimental group/condition, given as a discrete number and unit of measurement
☐	☒	A statement on whether measurements were taken from distinct samples or whether the same sample was measured repeatedly
☐	☒	The statistical test(s) used AND whether they are one- or two-sided<br>*Only common tests should be described solely by name; describe more complex techniques in the Methods section.*
☒	☐	A description of all covariates tested
☐	☒	A description of any assumptions or corrections, such as tests of normality and adjustment for multiple comparisons
☐	☒	A full description of the statistical parameters including central tendency (e.g. means) or other basic estimates (e.g. regression coefficient) AND variation (e.g. standard deviation) or associated estimates of uncertainty (e.g. confidence intervals)
☐	☒	For null hypothesis testing, the test statistic (e.g. *F*, *t*, *r*) with confidence intervals, effect sizes, degrees of freedom and *P* value noted<br>*Give P values as exact values whenever suitable.*
☒	☐	For Bayesian analysis, information on the choice of priors and Markov chain Monte Carlo settings
☒	☐	For hierarchical and complex designs, identification of the appropriate level for tests and full reporting of outcomes
☐	☒	Estimates of effect sizes (e.g. Cohen's *d*, Pearson's *r*), indicating how they were calculated

Our web collection on statistics for biologists contains articles on many of the points above.

Software and code

Policy information about availability of computer code

Data collection	10x Genomics Chromium Single Cell System V2, Illumina MiSeq, Illumina NovaSeq6000, Celeris Rodent Electrophysiology System, Scout Recording System, Odyssey XF Imaging System, Keyence BZ-X810
Data analysis	GraphPad Prism 9, Microsoft Excel 2016, Espion V6, Matlab R2019b, Cell Ranger 5.0.1, Seurat V3.1, ImageJ

For manuscripts utilizing custom algorithms or software that are central to the research but not yet described in published literature, software must be made available to editors and reviewers. We strongly encourage code deposition in a community repository (e.g. GitHub). See the Nature Portfolio guidelines for submitting code & software for further information.

Data

Policy information about availability of data

All manuscripts must include a data availability statement. This statement should provide the following information, where applicable:

- Accession codes, unique identifiers, or web links for publicly available datasets
- A description of any restrictions on data availability
- For clinical datasets or third party data, please ensure that the statement adheres to our policy

The main data supporting the results of this study are available within the paper and in the Supplementary Information. The deep-sequencing data generated in this study have been deposited in the Sequence Read Archive under accession number PRJNA739996 [https://www.ncbi.nlm.nih.gov/bioproject/739996]. Source data are provided with this paper.

Field-specific reporting

Please select the one below that is the best fit for your research. If you are not sure, read the appropriate sections before making your selection.

☒ Life sciences ☐ Behavioural & social sciences ☐ Ecological, evolutionary & environmental sciences

For a reference copy of the document with all sections, see nature.com/documents/nr-reporting-summary-flat.pdf

Life sciences study design

All studies must disclose on these points even when the disclosure is negative.

Sample size	No sample-size calculation was used to predetermine sample sizes. Sample sizes for mice were typically 6–9 mice per cohort, as this is standard for mouse studies. Sample sizes for mice eyes were also typically 8 eyes per cohort, with the exception of the off-target analysis (n = 3) and single-cell RNA seq analysis (n = 4). The sample size for the in vitro cell study was 3–6 replicates per cohort.<br>These sample sizes have been chosen in accordance with the standards of the field, which have been sufficient to draw conclusions and to achieve statistical significance.
Data exclusions	Mice with poor subretinal injections (that is, surgical injuries) were excluded. We pre-established these exclusion criteria and used them in the mouse studies.
Replication	The in vitro experiments were repeated independently at least three times, with similar results. All experiments involving animals were performed in at least three independent mice, with reproducible results
Randomization	The animals were randomly assigned to control and experimental groups. For all studies, samples and organisms were randomly allocated to the experimental groups.
Blinding	Deep-sequencing analysis were performed blindly. All other experiments were not blinded given that subsequent experiments were carried by the same researchers from the beginning.

Reporting for specific materials, systems and methods

We require information from authors about some types of materials, experimental systems and methods used in many studies. Here, indicate whether each material, system or method listed is relevant to your study. If you are not sure if a list item applies to your research, read the appropriate section before selecting a response.

Materials & experimental systems

n/a	Involved in the study	
☐	☒	Antibodies
☐	☒	Eukaryotic cell lines
☒	☐	Palaeontology and archaeology
☐	☒	Animals and other organisms
☒	☐	Human research participants
☒	☐	Clinical data
☒	☐	Dual use research of concern

Methods

n/a	Involved in the study	
☒	☐	ChIP-seq
☒	☐	Flow cytometry
☒	☐	MRI-based neuroimaging

Antibodies

Antibodies used	Mouse anti-RPE65 (in-house), rabbit anti-beta actin (Cell Signaling Technology, 4970S), mouse anti-Cas9 (Invitrogen, MAI523519, clone 7A9), goat anti-mouse IgG-HRP (Cell Signaling Technology, 7076S), goat antirabbit IgG-HRP (Cell Signaling Technology, 7074S), goat anti-S-opsin (custom-made by Bethyl Laboratories), cone arrestin (Millipore Sigma, AB15282), rabbit anti-M-opsin (Novus Biologicals, NB110-74730) , Alexa Fluor 488 donkey anti-goat IgG (Abcam, ab150129), Alexa Fluor 647 donkey anti-rabbit IgG (Abcam, 150075).
Validation	Each antibody was validated for species and application, as appropriate, on the manufacturer's website, as supported by relevant citations on the product pages. In-house produced RPE65 antibody has been validated and used in many publications (Choi, E.H., et al. Insights into the pathogenesis of dominant retinitis pigmentosa associated with a D477G mutation in RPE65. Human molecular genetics 27, 2225–2243 (2018)). Cas9 antibody and beta actin were validated by several publications including Suh et al., Nat Biomed Eng, 2021. S-opsin and M-opsin antibodies were validated by a previous paper (Leinonen et al., Invest Ophthalmol Vis Sci, 2019). For cone arrestin, the website states "Anti-Cone Arrestin, Cat. No. AB15282, is a highly specific rabbit polyclonal antibody that targets Arrestin-C and has been tested in Immunohistochemistry (Paraffin) and Western Blotting."

Eukaryotic cell lines

Policy information about cell lines

Cell line source(s)	NIH3T3, Phoenix-Eco, and HEK293T/17 were obtained from ATCC.
Authentication	NIH3T3, Phoenix-Eco, and HEK293T/17 were authenticated by ATCC. No further authentication was performed.
Mycoplasma contamination	NIH3T3, Phoenix-Eco, and HEK293T/17 were negative for mycoplasma contamination.
Commonly misidentified lines (See ICLAC register)	No commonly misidentified cell lines were used.

Animals and other organisms

Policy information about studies involving animals; ARRIVE guidelines recommended for reporting animal research

Laboratory animals	C57BL/6J, B6(A)-Rpe65 rd12/J mice, Gnat1-/-, 4–10 weeks-old and mixed gender.<br>The mice were housed at the animal facility of the University of California Irvine in a 12-hour-light (<10 lux) / 12-hour-dark cyclic environment. The temperature ranged from 75°F to 76°F, and the humidity from 30% to 40%.
Wild animals	The study did not involve wild animals.
Field-collected samples	The study did not involve samples collected from the field.
Ethics oversight	All animal procedures were approved by the Institutional Animal Care and Use Committee (IACUC) of the University of California, Irvine, and were conducted in accordance with the Association for Research in Vision and Ophthalmology Statement for the Use of Animals in Opthalmic and Visual Research.

Note that full information on the approval of the study protocol must also be provided in the manuscript.
